# The Potential Role of Iron Homeostasis and Ferroptosis in Exercise Nutrition and Health

**DOI:** 10.3390/nu18010139

**Published:** 2026-01-01

**Authors:** Qi Wang, Ruiyang Gao, Kongdi Zhu, Huilong Qiu, Jiaqiang Huang, Xia Zhang

**Affiliations:** 1 Institute of Artificial Intelligence in Sports, Capital University of Physical Education and Sports, Beijing 100191, China; 00020240005@cupes.edu.cn; 2 Department of Nutrition and Health, China Agricultural University, Beijing 100193, China; 15611678907@163.com (R.G.); zkd15938702692@163.com (K.Z.); qiucau2580@163.com (H.Q.)

**Keywords:** trace element iron, iron homeostasis, ferroptosis, exercise, nutrition

## Abstract

Iron is an essential trace element that participates in multiple physiological processes, including oxygen transport, electron transfer, DNA synthesis, and red blood cell production. Iron loss is particularly severe among athletes, so maintaining iron homeostasis is crucial for sports nutrition and health. Excess iron, iron deficiency, and ferroptosis can lead to muscle disorders and health issues, including sarcopenia, muscular atrophy, myocardial fibrosis, skeletal muscle injury, cardiovascular disease, and metabolic disorders. Maintaining iron homeostasis within physiological limits is essential for athletes to sustain high-intensity performance and accelerate recovery. Therefore, a comprehensive review of the effects of iron homeostasis and ferroptosis on muscle health is significant for identifying potential therapeutic targets and developing new disease treatment and prevention strategies. This paper systematically reviews research progress on targeted therapies for iron overload and ferroptosis in muscle diseases, clarifies the impact of iron on athletes’ physiological functions and competitive performance, and explores the potential application of iron in precision nutritional regulation. It aims to provide new insights for preventing exercise-induced muscle injury, myocardial damage, and overtraining syndrome in athletes.

## 1. Introduction

The human body contains a wide variety of elements. Those present at levels below 0.01% of body weight are typically classified as trace elements. They play a vital role in maintaining health by supporting growth and development, energy metabolism, and immune regulation [1]. In 1996, the World Health Organization (WHO) identified eight essential trace elements for humans: iron (Fe), iodine (I), zinc (Zn), selenium (Se), copper (Cu), molybdenum (Mo), chromium (Cr), and cobalt (Co) [2,3]. Trace elements exist in extremely small quantities within the human body, yet they play decisive roles in metabolic processes and physiological functions. Iron, for example, is a vital micronutrient required for numerous biological processes, including DNA synthesis, enzymatic reactions, and mitochondrial energy metabolism [4]. Ferritin plays a crucial role in maintaining iron homeostasis in the human body. Research indicates that adults require approximately 20 mg of iron daily for heme synthesis, which indirectly promotes hemoglobin production and participates in cellular metabolic activities [5,6]. Iron deficiency (ID) is a major contributor to the global disease burden, with anemia being one of its consequences [7]. ID anemia is defined as low hemoglobin levels resulting from depleted iron stores. The World Health Organization (WHO) has identified iron deficiency anemia as the most prevalent nutritional deficiency worldwide, affecting approximately 1.2 billion people, or 30% of the global population [8,9]. For athletes in particular, ID significantly impacts athletic performance. Researchers believe that high-intensity training leads to the loss of trace elements through sweat and urine, making athletes more susceptible to the effects of trace element imbalance [10,11]. In particular, ID inhibits red blood cell production and reduces hemoglobin levels, which directly weakens the body’s aerobic metabolism and consequently diminishes athletic endurance [12]. Additionally, ID disrupts metabolic processes, impairing motor coordination in athletes. Studies reveal that exercise intensity significantly influences serum Fe levels, and professional middle- and long-distance runners exhibit markedly lower serum Fe concentrations than amateur athletes [13]. Recent clinical research indicates an association between iron deficiency and muscle strength. ID is significantly correlated with reduced muscle strength in patients undergoing maintenance hemodialysis (MHD) [14]. Decreased exercise capacity and muscle mass in heart failure patients are also linked to iron deficiency [15]. In both acute and chronic gout patients, ID leads to reduced muscle mass without improving skeletal muscle growth [16,17]. RNA-seq analysis indicates that iron deficiency primarily affects genes involved in glycolytic energy metabolism, cell cycle regulation, and apoptosis in muscle cells [18]. Iron is essential for maintaining skeletal muscle energy metabolism and overall function. It plays a critical role in oxygen uptake, transport, storage, and oxidative metabolism in both cardiomyocytes and skeletal muscle cells [19,20]. Maintaining muscle health is essential for normal physiological function and long-term well-being in humans. Maintaining muscle health is essential for optimal physiological function and long-term wellness. It impacts physical strength, metabolic levels, the ageing process, and quality of life. Muscle strength and endurance are especially important for athletic training and competitive performance. Scientific research on the precise regulation of iron nutrition is of vital physiological and nutritional significance. It ensures blood oxygen transport, muscle energy metabolism, and the orderly circulation of iron throughout the body. This research also prevents pathological issues associated with imbalanced iron metabolism.

Therefore, the relationship between iron and athletic health is a topic worthy of attention in the field of medicine. This paper explores the relationship between iron and human exercise and muscle health, revealing its connection to disease prevention and treatment. This paper reviews the physiological functions of iron metabolism in muscle tissue. It discusses how iron homeostasis and ferroptosis affect muscle health. Additionally, it summarizes the current progress of research on targeted ferroptosis therapies and emphasizes the effects of iron deficiency and supplementation on athletes’ physiological and competitive performance. Finally, the paper discusses the development prospects of precision iron nutrition regulation in sports nutrition, aiming to enhance public understanding of iron’s role in athletic health. It encourages athletes to supplement iron through functional foods in a targeted manner and provides scientific direction for future therapies that address muscle disorders.

## 2. Iron Homeostasis and Importance in Maintaining Muscle Health

### 2.1. Iron Homeostasis and Function

Fe is an essential trace element for the human body. Healthy adults have a total iron content ranging from 3 to 5 g, 70% of which is heme iron [21]. Heme iron is found in hemoglobin and myoglobin. The rest of the iron is stored as ferritin or ferritin-containing heme in hepatocytes and reticuloendothelial cells. Fe is transported throughout the body bound to transferrin. Ferritin is the primary intracellular form of iron storage and a crucial component of cellular iron homeostasis [22]. A healthy adult can obtain 1–2 mg of iron daily from the diet, whereas hemoglobin synthesis requires 20–25 mg daily [23,24]. To compensate for this deficit, the body maintains a dynamic equilibrium of iron through continuous absorption, utilization, storage, and recycling—a process termed iron homeostasis [25]. Disruption of this equilibrium can lead to disorders related to iron metabolism, including iron overload and deficiency diseases [26]. Iron homeostasis plays a critical role in vital processes such as erythropoiesis, muscle energy metabolism, cell cycle regulation, hormone production, immune system function, heme synthesis, DNA replication and repair, brain development, ageing, and cytochrome formation.

**Figure 1:**
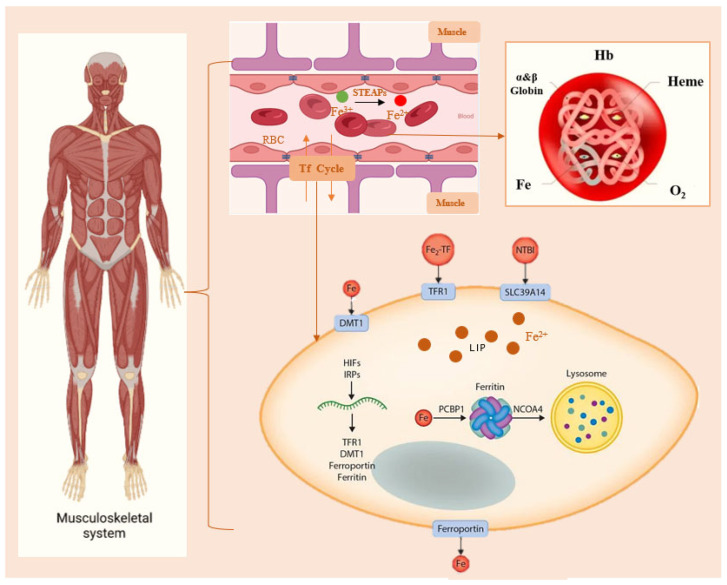
Regulation of Iron Metabolism in Muscle. Within the body’s muscular system, Fe in the blood delivers oxygen by binding to Hb. Then, Fe^2+^ enters muscle cells via Tf, Fe^2+^ is typically stored in the cytoplasm or lysosomes. When blood iron levels are insufficient, muscle cells release Fe^2+^ again. STEAPs: Six-transmembrane epithelial antigen of prostates. Hb: Hemoglobin. Tf: transferrin. DMT1: divalent metal transporter 1. TFR1: transferrin receptor protein 1. NTBI: non-transferrin-bound iron. SLC39A14: metal transporter proteins. LIP: labile iron pool. PCBP1: Poly(rC)-binding protein 1. NCOA4: Nuclear receptor coactivator 4.

The muscle system is one of the largest and most important organs in the human body. The muscular system controls skeletal movement, cardiovascular function, and digestive organ activity. Its health status spans the entire lifespan. Exercise is a key external factor that stimulates muscle growth and maintains muscle function. The musculoskeletal system is essential for movement and support. Muscles attach to bones to maintain standing and sitting posture and to cushion joint pressure during activities such as walking, running, and jumping [27]. Additionally, muscles serve as primary metabolic sites, and moderate exercise enhances overall metabolic efficiency. Physical activity improves blood circulation to the muscles, delivering increased oxygen and nutrients to muscle cells and thereby boosting muscular endurance. Maintaining muscle health significantly delays the ageing process and prevents muscle loss [28]. Among the elderly population, muscle loss can easily lead to a decline in muscle movement quality and functional deterioration. The two primary mechanisms of muscle mass loss are muscle fibre atrophy and loss of muscle fibres [29]. Iron plays a crucial role in maintaining muscle health. Research indicates that higher serum iron levels reduce the risk of sarcopenia, and emerging evidence suggests a strong association between ferroptosis and muscle wasting [30,31]. Fe is the core component of hemoglobin and is responsible for transporting oxygen from the blood to muscle cells. This ensures a sustained energy supply during prolonged exercise, which prevents endurance decline and fatigue caused by hypoxia. Thus, optimal muscle efficiency is maintained (Figure 1). It is critical to maintain iron balance within muscles; both deficiency and excess can harm the body.

### 2.2. The Effects of Iron Overload on Muscle Health

Iron is a beneficial component of the human body and a major dietary micronutrient that participates in key cellular functions and metabolic processes. However, excessive iron increases the production of reactive oxygen species (ROS), which can lead to cellular dysfunction or death, tissue damage, and organ disease. Sarcopenia, the progressive loss of skeletal muscle mass and strength, is highly correlated with iron overload; it exhibits high prevalence among the elderly and significantly impairs quality of life [32]. A study examining the relationship between muscle iron content, fat infiltration, and age in 198 subjects revealed that muscle iron content and fat fraction increase with age while grip strength decreases, findings associated with sarcopenia [33]. Furthermore, multiple studies have documented age-related iron accumulation in skeletal muscle. Excess iron may contribute to skeletal muscle damage or atrophy, and elevated serum ferritin levels have been observed in patients with sarcopenia [34,35,36]. Research into oxidative stress in space induced by iron overload indicates that superoxide in cells can promote the conversion of Fe^3+^ to Fe^2+^, thereby catalyzing the iron electron transfer cycle [37,38]. Increased serum ferritin concentrations—an indicator of iron storage—following both short- and long-duration spaceflight suggest that elevated iron storage may induce oxidative damage [39,40]. Iron overload not only increases the risk of ferroptosis but also impairs muscle stem cells, thereby affecting skeletal muscle repair and regeneration. Skeletal muscle iron overload exacerbates muscle atrophy. Research indicates that iron overload induces muscle atrophy in patients with gastric cancer cachexia. Differential gene analysis suggests that ferritin, iron and oxidative stress may be associated with skeletal muscle wasting. Gastric cancer patients with elevated ferritin levels are more prone to muscle atrophy symptoms [41]. In recent years, iron has emerged as an independent factor affecting muscle and skeletal muscle physiology. Iron overload is associated with muscle health issues stemming from secondary oxidative damage, which is a primary concern for astronauts. Excess iron accumulates in muscle cells. Once ferritin becomes saturated, it forms hemosiderin, which damages mitochondria and triggers muscle soreness, weakness, and rhabdomyolysis. Concurrently, aerobic respiration in muscles is impaired, leading to reduced energy production.

### 2.3. The Impact of Iron Deficiency on Muscle Health

Iron deficiency is a common nutritional disorder, typically caused by inadequate dietary intake of iron, impaired absorption of iron, or excessive loss of iron. It readily leads to structural alterations and functional impairments in tissues and organs, triggering a variety of physiological manifestations. In terms of muscle function, iron deficiency leads to reduced strength, endurance and reaction speed. With regard to bone health, intracellular iron deficiency can disrupt the activity and homeostasis of osteocytes and osteoblasts, resulting in an imbalance in bone homeostasis and ultimately leading to bone loss or osteoporosis [42]. The mineral element iron helps to maintain myocardial integrity and energy metabolism, exerting a wide range of effects on cardiac function [43]. Furthermore, there are pathophysiological interrelationships between iron deficiency anaemia, renal dysfunction and heart failure; anaemia reduces tissue oxygenation, exacerbating heart failure symptoms [44]. A study examining the impact of iron metabolism on rhabdomyosarcoma (RMS) tumour growth demonstrated that iron supplementation effectively inhibits tumour proliferation. Maintaining elevated iron levels within cancer cells accelerates metabolic activity [45]. Additionally, restless legs syndrome (RLS) is a clinically prevalent neurogenic sensory-motor disorder. Evidence suggests that non-anaemic iron deficiency is a common predisposing factor for RLS and is potentially associated with alterations in mitochondrial energy metabolism and hypoxia-inducible factor expression [46]. All mammalian cells require iron, which binds to plasma transferrin receptors. Transferrin-mediated iron assimilation is critical for skeletal muscle metabolism, and studies indicate that intramuscular iron deficiency can cause severe systemic metabolic disorders [47]. However, iron deficiency can also cause muscle soreness. Iron is involved in haemoglobin synthesis, which aids the transport of oxygen to tissues throughout the body. When iron is insufficient, muscles may be forced into anaerobic metabolism due to hypoxia and impaired energy metabolism. This leads to increased lactic acid accumulation and symptoms such as muscle soreness and fatigue, which are particularly noticeable after intense exercise [48]. In summary, maintaining iron homeostasis relies on the proper functioning of iron regulatory mechanisms throughout the body. Further investigation is warranted into the iron metabolic pathways within muscle tissue and their underlying molecular biological mechanisms. As both iron deficiency and overload are detrimental to muscle health, strict regulation of iron homeostasis in muscle tissue is essential for appropriate iron metabolism.

## 3. Ferroptosis and Targeted Therapy

### 3.1. Ferroptosis Overview

**Figure 2:**
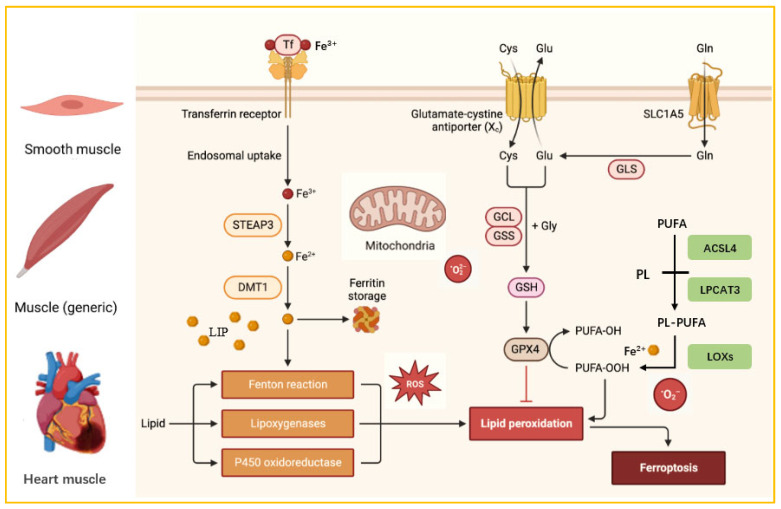
Three Metabolic Pathways of Cellular Ferroptosis. STEAP3: Six-transmembrane epithelial antigen of prostate 3. DMT1: divalent metal transporter 1. SLC1A5: System Xc− consists of a solute carrier family 1 member 5. PUFA: polyunsaturated fatty acid. PL: phospholipid. ACSL4: acyl-CoA synthetase long-chain family member 4. LPCAT3: lysophosphatidylcholine acyltransferase 3. LOXs: lipoxygenases.

Ferroptosis is a form of regulated cell death that is iron-dependent and characterized by the accumulation of lipid peroxides, heightened oxidative stress and the depletion of cellular antioxidant defences [49]. This unique cell death pattern is typically triggered by oxidative stress caused by iron overload and initiated by iron-dependent lipid peroxidation of phospholipids [50]. It is also regulated by multiple cellular metabolic pathways, including those responsible for redox homeostasis, mitochondrial function, amino acid metabolism, lipid metabolism, and glucose metabolism [51]. Ferroptosis generally follows three primary pathways regulated by lipid metabolism, iron homeostasis, and redox balance (Figure 2). Firstly, lipids and lipid peroxidation play a pivotal role in the progression of ferroptosis. Cells accumulate hydroxyl radicals and reactive oxygen species (ROS) through the Fenton reaction with hydrogen peroxide. These ROS then oxidize the polyunsaturated fatty acids (PUFAs) in the cell membrane, forming lipid peroxides and disrupting membrane structural integrity. This process also attacks DNA and proteins, triggering ferroptosis [52,53]. Secondly, iron overload is the main cause of ferroptosis, and maintaining iron homeostasis helps to protect cells against it. When there is an excess of iron, transferrin receptors become saturated, which accelerates iron turnover. Excess iron accumulates in unstable iron pools, triggering the Fenton reaction within cells and generating excessive ROS [54,55]. Finally, an imbalance between cellular antioxidant defence systems and oxidative stress can also trigger ferroptosis. Glutathione peroxidases and transferases play a role in the antioxidant defence against ferroptosis. Glutathione (GSH) acts as a cofactor for GPX4 to promote the reduction in phospholipid peroxides and mitigate oxidative damage induced by ferroptosis. Consequently, reduced intracellular GSH levels may lead to ferroptosis [56,57,58]. The pathways that govern iron uptake, utilization, storage, conversion and excretion within cells are crucial in regulating ferroptosis.

### 3.2. The Role of Ferroptosis in Muscle Diseases

It is well known that maintaining normal muscle physiology requires iron homeostasis. Compared to other tissues, muscle tissue requires more energy to maintain its active state, possesses a large number of mitochondria, and is therefore a major source of reactive oxygen species (ROS), which makes muscle cells more susceptible to ferroptosis [59,60]. Age-related sarcopenia induced by ferroptosis has been associated with iron overload, which has been detected in sarcopenic patients and in the skeletal muscle of young mice [61]. In aged rats, elevated tissue iron levels were observed alongside reduced skeletal muscle mass [62]. Ferroptosis triggers cardiomyopathy and heart failure. Knocking out ferritin in mice leads to excessive iron accumulation in cardiomyocytes, resulting in heart failure at an early embryonic stage [63]. Furthermore, feeding mice an iron-rich diet causes cardiac injury and hypertrophic cardiomyopathy, exhibiting hallmark features of ferroptosis [64]. Ferroptosis may be associated with diabetic cardiomyopathy. Studies indicate that inhibiting ferroptosis can reduce myocardial damage in people with diabetes. Activation of the renin receptor induces ferritinophagy, thereby promoting ferroptosis and contributing to diabetic cardiomyopathy [65,66]. Research shows that ferroptosis causes injury to vascular smooth muscle, with iron accelerating calcification in human aortic smooth muscle cells. Furthermore, ferroptosis has been linked to vascular calcification in patients with chronic kidney disease (CKD) [67,68]. A 48-year-old male patient with coronavirus disease 2019 (COVID-19) exhibited myocardial cell degeneration and necrosis in cardiac tissue, alongside severe lipid peroxidation. This reflects myocardial inflammation associated with ferroptosis [69]. Additional studies indicate that muscle inflammation triggers KMT5A-mediated monomethylation of lysine 20 on histone H4 (KMT5A-H4K20me1). This process promotes the epigenetic silencing of muscle stem cells (MuSCs) and ultimately induces ferroptosis, causing aged muscle stem cells to undergo a rusty meltdown [70]. Accumulating evidence suggests that iron overload and ferroptosis are key mechanisms underlying muscle diseases and could be targeted to prevent progression.

### 3.3. Research Progress on Targeted Therapy for Ferroptosis

Diseases associated with ferroptosis have been identified in people of all ages, including infants, toddlers, teenagers, adults and the elderly. These diseases include heart and lung disease, digestive and immune system disorders, cancer and age-related macular degeneration. As we age, increased iron accumulation in the body readily induces ferroptosis, heightening susceptibility to diseases associated with hypoxia, ischaemic muscle injury, skeletal muscle damage, muscle wasting and atrophy. A recent article in the journal Nature reported that knocking out the ferroptosis suppressor protein 1 (FSP1) in mice increased lipid peroxidation levels and significantly suppressed tumour formation, indicating that lung cancer is highly sensitive to ferroptosis [71]. In a clinical trial lung cancer model, the research team demonstrated that pharmacological inhibition of FSP1 significantly treated lung cancer. Additionally, intratumoral monotherapy with a selective FSP1 inhibitor effectively suppressed melanoma growth in lymph nodes, indicating that targeting FSP1 in lymph nodes has great potential for preventing melanoma progression [72]. Ferroptosis inhibitors have been widely used to slow and repair muscle diseases, demonstrating controllable safety in clinical settings. Targeting ferroptosis opens new avenues for treating ferroptosis-related diseases. This paper outlines several research strategies (Table 1). A recent study indicates that numerous novel targets have been identified for treating myocardial injury and disorders of iron metabolism by modulating ferroptosis. These include Yin Yang 1 (YY1) and Yin Yang 2 (YY2), autotaxin (ENPP2), heat shock factor 1 (HSF1), protein arginine methyltransferase 4 (PRMT4) and circRNA. These targets regulate iron metabolism and lipid peroxidation, protect cardiomyocytes from ferroptosis and alleviate myocardial injury [73]. Although targeted therapeutic approaches have limited utility in muscle diseases, they facilitate the elucidation of ferroptosis mechanisms in these conditions. In recent years, novel targeted therapeutic technologies have advanced rapidly. Among these are fluorescent probes that can monitor key targets during ferroptosis, facilitating the diagnosis of muscular diseases and the development of therapeutic agents [74]. The photochemical activation of membrane lipid peroxidation (PALP) shows promise in the rapid screening and assessment of cancer patients’ sensitivity to ferroptosis [75]. Photodegradation-targeting chimera (PDTAC) can specifically trigger ferroptosis in immune cells by inducing GPX4 dysfunction, with potential applications in cancer immunotherapy [76]. Two recently identified forms of cell death regulation, ferroptosis and copper-mediated apoptosis, have been studied for targeting gastrointestinal cancers. Understanding the relationship between ferroptosis and copper-mediated apoptosis could lead to novel, synergistic therapies against gastrointestinal malignancies [77]. Overall, identifying the regulatory patterns of key ferroptosis targets is crucial for designing novel targeted therapies. Current research on ferroptosis in muscle diseases and its pathophysiological mechanisms has received considerable attention.

## 4. The Connection Between Trace Element Iron and Athletes

### 4.1. Effects of Iron on Athletes’ Physiological Functions

Iron deficiency is the most common physiological disorder experienced by athletes, and disruption to iron homeostasis induced by exercise adversely affects training and athletic performance. A study of 14 female runners found that 10 of them developed iron deficiency after training, with iron depletion reaching a prevalence of 71% [85]. Ferritin and iron stores decreased during prolonged training and failed to recover within 10 days. During and after intense exercise, impaired oxygen transport and hypoxia-inducible factors decrease ferritin expression [86]. Another study found significantly reduced ferritin concentrations and haemoglobin levels in 18 female long-distance runners after eight weeks of endurance training [87]. A further study investigated iron levels in 2749 college athletes, defining iron deficiency as ferritin levels below 20 ng/mL for both genders. The results indicated iron deficiency in 33.1% of female athletes and 4.1% of male athletes [88]. These results suggest that female athletes are more susceptible to iron deficiency than male athletes within athletic populations. Athletes experience a decrease in serum protein concentration and iron levels after exercise, which indirectly indicates increased iron requirements. Intense physical activity accelerates iron loss through sweat and urine, and in severe cases, this can lead to anaemia, causing generalized weakness and indirectly impacting athletic performance [89]. High-intensity aerobic and anaerobic training sessions often result in significant fatigue among athletes, particularly professionals [90]. When training intensity exceeds the body’s capacity for self-regulation and recovery, the effectiveness of training diminishes significantly. Athletes may experience lethargy, muscle weakness, fatigue and exhaustion [91]. Beyond adequate rest, physical stretching and the body’s self-repair mechanisms, timely iron supplementation is crucial to promote recovery of athletic function. Additionally, ensuring an adequate iron intake helps to maintain the transport capacity of red blood cells, thereby enhancing oxygen delivery to tissues. This prevents athletes from experiencing diminished performance due to an insufficient oxygen supply. Iron nutrition also significantly improves bodily tissues, further increasing the body’s resistance to fatigue.

### 4.2. Effects of Fe on Athletes’ Performance

**Figure 3:**
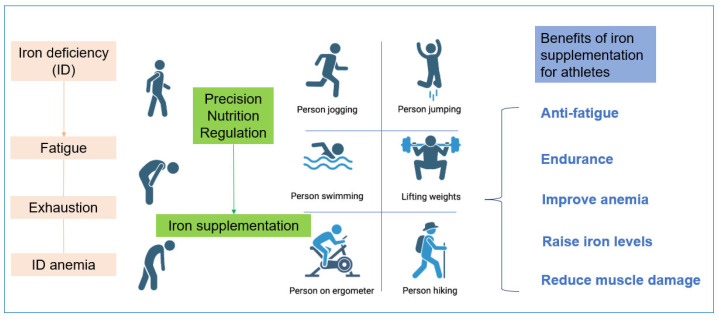
The Benefits of Fe-Precision Nutritional Regulation for Athletes.

Iron deficiency is a widespread nutritional problem. Physical exercise can lead to significant iron loss, resulting in a relatively high prevalence of iron deficiency among athletes. Around 57% of female athletes and 31% of male athletes experience issues with iron depletion [92]. The most common symptom is impaired aerobic endurance performance, and adolescent and female athletes are particularly susceptible to iron deficiency. For fitness enthusiasts, an adequate supply of iron and oxygen forms the foundation for enhancing athletic performance. Iron accelerates haemoglobin synthesis, enabling more efficient oxygen transport during exercise and boosting endurance and stamina in aerobic activities such as running, swimming and cycling. It also reduces fatigue during strength training and facilitates faster recovery (Figure 3) [90]. During endurance training, athletes typically push their physical limits, placing excessive strain on the body. Insufficient iron intake leading to deficiency can severely impair athletic performance [93]. At the beginning and end of a 10-week pre-season training period, iron levels were measured in 30 female Australian rugby players, suggesting that iron deficiency may impair athletic strength performance [94]. Another study involving 669 athletes across 16 sports (aged 13–47) showed that iron deficiency reduces endurance performance by 3–4% [95]. Oral iron supplementation can prevent iron depletion. Taking 325 mg of ferrous sulphate daily for 11 weeks enhanced strength performance in female volleyball players during the competitive season, including exercises such as bench press, deadlift, half squat and pull-up [96]. Additionally, taking 30 mg of elemental iron in the form of ferrous sulphate daily for six weeks significantly improved iron status and endurance in iron-deficient, non-anaemic male and female subjects [97]. Athletes require optimal iron levels to maintain peak performance throughout the training cycle, and precision nutritional iron supplementation offers multiple potential benefits for athletes across various disciplines.

## 5. The Development Prospects of Iron in Sports Nutrition Products

Sports nutrition products are dietary supplements that can improve health and support normal bodily functions, as well as maintain endurance and strength. Athletes who take trace elements such as iron may enhance their athletic training performance. However, both iron deficiency and overload can adversely affect physiological states to varying degrees, preventing optimal training outcomes [98]. Therefore, during supplementation with trace elements, intake must be strictly controlled according to individual needs to ensure these micronutrients effectively enhance athletic performance and reaction times. According to the 2023 edition of China’s Dietary Reference Intakes for Nutrients, the recommended nutrient intake (RNI) for adult males is 12 mg per day, and for females it is 18 mg per day [99]. In sports science, precision nutrition regulation has emerged as a key focus in interdisciplinary sports nutrition research. This innovative approach involves tailoring nutritional strategies to optimize athletic performance precisely [100]. This enables the creation of personalized dietary plans for athletes and ensures the scientific and efficient supplementation of nutrients. According to market analysis, the global sports nutrition market was valued at $49.6 billion in 2024. It is projected to reach $53.27 billion by 2025 and $94.3 billion by 2033, representing a compound annual growth rate (CAGR) of 7.4% during the forecast period (2025–2033), (https://straitsresearch.com/zh/report/sports-nutrition-market (accessed on 11 November 2025)). Due to the varying intensity and form of daily training among athletes in different sports, iron loss in the body can differ significantly. Chokeberry (poly)phenol-rich supplementation may be effective in enhancing the redox balance of athletes, and supplementation with lyophilized black chokeberry extract improves the performance and antioxidant status of serum in young football players [101,102]. Healthy diet recommendations, developing iron-supplemented sports nutrition products and scientifically enhancing overall physical fitness and athletic performance. Energy bars and gels enriched with animal-derived heme iron (extracted from meat, liver, and blood products) are designed for rapid replenishment without digestive burden and are ideal for endurance and explosive power athletes, such as those in middle/long-distance running, cycling, basketball, and soccer. Additionally, plant-based products derived from black beans, nuts, and vegetables should be developed to enhance non-heme iron absorption for vegetarian athletes [103]. However, excessive iron supplementation poses risks by disrupting the metabolic homeostasis of iron absorption, transport, and storage. These issues, which include oxidative damage and tissue deposition, cannot be overlooked in iron metabolism research [104,105]. These findings also provide key evidence for the clinical and nutritional application of appropriate iron supplementation. The development of iron-supplemented foods for athletes will be closely linked to advancements in sports nutrition research and food processing technologies, as well as the growing demand for personalized nutrition.

## 6. Conclusions

Overall, iron homeostasis is the collective metabolic balance of iron in the body and is fundamental to athletes’ health and performance. Ferroptosis, a form of programmed cell death driven by iron-dependent lipid peroxidation, is a novel mechanism underlying potential exercise-induced injuries. Disorders of iron metabolism and ferroptosis play a key role in various muscle diseases, including sarcopenia, cardiomyopathy, muscular dystrophy and amyotrophy. Dietary iron supplementation can rapidly increase serum ferritin levels. However, the potential of targeting ferroptosis to treat muscle diseases is limited. Precision medicine approaches are highly challenging due to the complexity and uncertainty of intercellular interactions, and current clinical trials are incomplete. For example, the long-term administration of ferroptosis-targeting agents such as DFO, DFX and NAC can result in irreversible damage to other organs. Nevertheless, targeting ferroptosis has paved the way for a new therapeutic approach to human diseases.

Targeting ferroptosis is essential in the treatment of muscle diseases. However, several questions remain unanswered: Firstly, does intracellular iron metabolism affect immune cell function? Secondly, how can the iron dosage-response relationship within cells be precisely determined? Thirdly, how can we identify early diagnostic markers for ferroptosis in cells? Therefore, future research should focus on discovering novel biomarkers for early diagnosis. Elucidating the mechanisms linking iron supplementation dosage to exercise adaptation will enable the development of personalized nutritional strategies. These strategies could then be used to develop tailored iron supplementation protocols for different athletes, achieving precise iron regulation in sports nutrition. Finally, targeted intervention methods should be developed to accelerate iron recovery in athletes following high-intensity training. Research into trace elements and sports nutrition will directly promote deeper integration and transformation within exercise physiology, sports medicine and sports nutrition.

## Figures and Tables

**Table 1:** Methods and Effects of Targeting Ferroptosis.

Type of Disease	Targeted Drugs	Dosage	Treatment Cycle	Therapeutic Effect	References
Beta-thalassemia major	deferasirox (DFX) and deferoxamine (DFO) combination therapy	DFX 20–30 mg/kg daily; DFO 35–50 mg/kg on 3–7 days/week	12 months	The concentrations of plasma ferritin, hepatic iron, myocardial iron and non-ferritin-binding iron in the plasma are reduced.	[78]
Iron overload beta-thalassemia major	DFX and DFO stand-alone treatment	DFX 40 mg/kg daily; DFO 50–60 mg/kg on 5–7 days/week	24 months	The myocardial T2 signal intensity improved over a two-year period, while the hepatic iron concentration decreased.	[79]
Severe transfusional iron overload	DFX and DFO combination therapy	DFX 30.5 mg/kg per day; DFO 36.3 mg/kg per day	24 months	The T2 value increased from 7.2 ms at baseline to 9.5 ms after 24 months, indicating a reduction in myocardial and hepatic iron concentrations.	[80]
Skeletal muscle injury	Thiol-based antioxidant *N*-acetylcysteine (NAC)	20 mg NAC/kg in 3 daily dosages	8 days	Reduce inflammation and exercise-induced muscle damage. Improve skeletal muscle performance and inhibit intracellular, redox-dependent signalling pathways.	[81]
Acute myocardial infarction	N-acetyl cysteine (NAC)	intravenous injection, total dose 6000 mg (12 mL) of NAC	120 h	Prevention of Non-Thyroid Disease Syndrome. This is in patients with acute myocardial infarction.	[82]
Diastolic heart failure	Ubiquinol and D-ribose	600 mg of ubiquinol and 15 g of d-ribose per day	12 weeks.	Alleviate heart failure symptoms, reduce B-type natriuretic peptide and lactate levels, and increase ATP production.	[83]
Duchenne muscular dystrophy (DMD)	Idebenone	300 mg three times a day	52 weeks	It improves respiratory muscle function and reduces the loss of respiratory function. It is also safe and well tolerated.	[84]

## Data Availability

Links to publicly archived datasets analyzed or generated during the study.
